# Selections and Abstracts

**Published:** 1906-11

**Authors:** 


### SELECTIONS AND ABSTRACTS.

### Lesions Predisposing to Cancer.*

By M. B. Hutchins, M.D., Atlanta, Ga.

It is difficult to avoid bringing into a paper of this kind the cause of origin of cancerous growths, since cause and prior lesion are so intimately related. Without some sort of stimulus or disturbance of cell relation many of the lesions would never become malignant. Whether a group of cells has been left out of place in fetal life and ready for aberrant growth, or gets misplaced later, makes no great difference for the purposes of this paper, but the latter occurrence seems best to harmonize with the facts. That mechanical, thermic, actinic, chemical, and perhaps local toxic influences often seem the positive exciting cause of malignant development can not be doubted. What the element is in cell relations that holds the balance in so many cases and loses its control in a few, we do not know.

Of the skin lesions tending to cancerous development there are none that rank so high as the rough, horny, scale-crusted, irregular points and patches, varying from pinhead to all sizes and shapes seen on the face, neck and hands. These occur so frequently on the thin, red, florid skin of people who are either red-haired, or have the "red-haired" type of skin as to become a definite feature. The crusts are usually composed of small, horny cell masses; the skin beneath may show a bit tender, or it may be slightly thickened and irregular. Later, one or more points take on typical epitheliomatous changes, cell-growth, infiltration, erosion, crusting, fissuring, ulceration. Often there are numerous epitheliomata on the one subject; but a majority of people go through life with their "scaly spots" better or worse, without reaching the malignant stage; hence radical treatment for these must await such development.

*Read before the Medical Association of Georgia, at Augusta, April 20, 1906.

Closely related to the above, or occurring with it, is the oily, seborrheic skin with points, discs or patches developing in a greasy surface, and showing greasy scales and, sooner or later, erosions, new growth, finally the epithelioma in full development. These two kinds of skins and lesions more frequently show multiple epitheliomata. Warty, flat, roughened patches, as of the "senile skin," often form the starting-point of cancer. A persistent fissure of the lip may be the origin of epithelioma. Scars, as from burns, caustics, X-rays, incisions, boils, carbuncles and various injuries present a condition of cell displacement favorable to malignant growth.

A lesion from the pinch of a cracked and foul pipe-stem has caused death from cancer of the lip. Phimosis and balanitis at times result in epithelioma. An innocent-looking epulis may prove not a fibroma, but a sarcoma.

Lupus erythematosus has been known to degenerate into cancer. Lupus vulgaris scars often so change. An apparently innocent wart may become malign, as may keratoses from long continued use of arsenic. Keene* in his paper before the American Medical Association two years ago insisted on the latent dangers in and the removal of all moles, but was convinced of the impossibility of getting consent of the patient to such radical prophylaxis. My own observation is that few moles undergo cancerous change; but when they do, especially those of the pigmented type, their malignance and metastases may be horribly complete; their termination fatal.

The pigmentary type of malignant disease is the product of many lesions. A mole accidentally burned, and so being transformed into malignancy, is recorded by Crocker.† Johnson‡ reports cancer developing in a mole on the thigh; another on the back; another on the shoulder, and one on the abdomen. Following injury near the nail, a swelling, dark fluid discharge, pigmentary deposit in the growth—"melanotic whitlow"—may develop.

*The Journal A. M. A., July 9, 1904.

†Crocker: "Diseases of Skin," 1905.

‡Johnston: Jour. Cut. Dis., Feb. 1905.

"Malignant freckle" spot on foot or leg may end in pigmentary cancer. Melanotic rodent ulcer or melano-epithelioma is another form. Melonoma of the eye has its usual beginning in a choroidal pigmentary spot.

It is often impossible to diagnose the type of these growths accurately without the aid of the microscope. Metastases of pigmentary cells are often extensive, the lethal end certain. Early and free surgical removal offers the only cure.

Wilfred Fox* considers most cases of melanoma as nevocarcinoma. Johnston found both types, nevocarcinoma and nevosarcoma; Fox, at variance with Johnston, convicts the pigment of the chief activity in cancerous change.

Cases under X-ray treatment for epithelioma often show partial, possibly complete destruction of the original disease with extension, or new development of disease in the scar or its borders. Skiagraphic X-ray burns and the burns of X-ray workers have time and again gone on to epithelioma and death. *McLeod's case of a lupus-X-ray scar with cancer forming in its middle is striking.

Suppurative, broken-down sebaceous cysts, or even the unbroken cysts, may be transformed into cancer.

A small injury of the tongue from a jagged tooth, and continued irritation, have sufficed to bring on cancer of that organ. A similar development may follow a syphilitic gumma. So with leucoplasia, the white spots and bands called "smoker's patches." Constant irritation of a tonsillary, pharyngeal, or esophageal lesion may terminate in the same way. Many cases of cancer of the stomach follow an ulcer; many of the gall bladder succeed erosions from biliary calculi. Ovarian cysts may so degenerate. Uterine myoma is not always benign. Cervical scars from old lacerations frequently form the starting-point of cancer. Leucoplasia of the vulva often undergoes malignant change.

No adenoma of the breast is safe left in its place. The cicatrix from abscess of the breast has been sufficient to furnish the origin of cancer. Paget's disease of the nipple, which

*Fox: Brit. Journ. Derm., March, 1906.

may possibly be confused with eczema, is usually cancerous in nature and progression.

Sufficient examples have been given and time and space are already too far exhausted to permit of an extension of the list. In unnatural lesions and growths, in scars and irritated erosio s, in parts subjected to continued injury, we have a loss of cell balance, an absence of normal restraint, and the scene laid for the entrance of the unknown element that determines the onset of carcinoma, sarcoma or endothelioma, all malignant.

To urge on people the dangers in lesions and growths not yet malignant will often be of no avail. In many cases the malignant change may never take place, but every effort should be made to cure irritated lesions and to remove irritant agents in the simple cases; and no growth certainly progressing towards cancerous evolution should be left untreated. On the slightest occurrence of symptoms tending to these changes, instant and thorough treatment must be instituted. Often the simplest erosion, smallest scirrhus or most minute irritated mole or other growth may liberate cells that wander away to form metastases of the most formidable character. It is proper to watch some cases without interference, and criminally negligent or ignorant to advise the patient to "let alone" any lesion or growth already showing signs of malignancy.

I have used the term cancer in its broad sense, to cover all types of growth whose tendency is to progression, insidious diffusion, recurrence after treatment, certain death without treatment, often death in spite of treatment.

### REPORT OF CASES.

A few illustrative cases are here reported:

CASE 1.—Mr. Q., aged 59, cut with a razor a large, flesh-colored mole on his left cheek eight months before consulting me. The microscope showed epithelioma. It was excised. Permanent recovery.

CASE 2.—Mr. C. had a fungating and eroded pigmentary lesion on the top of left shoulder. There had been a dark mole there for years. It was irritated by suspenders. The man recovered after prolonged caustic treatment and protection.

CASE 3.—Mr. M., aged 66. A diagnosis of melanosarcoma of dorsum right foot was made. Forty years previously a black dot of pinhead size appeared just to the right of the great toe's extensor tendon. There was a slow peripheral growth for twenty-five or thirty years; then it increased more rapidly, especially in the last three or four years. When seen, the lesion was two by two inches in size, with various shades and degrees of pigmentation from brown to black or bluish. There was some thickening, and in parts it was finely papular. There were burning sensations when warm in bed. The man declined excision and received no treatment. About a year later part of the patch was elevated and exuding serum, but still there was no metastasis. The man died within a year or two of general diffusion of the disease.

CASE 4.—Mr. A., aged 77, had had more or less epitheliomatous trouble for fifteen years. Skin of face, neck and hands showed many scaly points and spots and scars of excisions, etc. When seen he had a fungating epithelioma of the left zygomatic malar arch and bone, growing to bones beneath. On the right maxillary ramus there was another large one. On the left lower eyelid there was a small hard epithelioma with erosion and crusting. Treatment was purely palliative. The case was incurable. Cases like the above and the one to follow are the more numerous in records.

CASE 5.—Dr. —, aged 53. Usual florid type. There was in a crusted lesion on the left cheek a typical cutaneous horn, beneath—an epithelioma, surrounded by greasy scales. On the end of the nose there were waxy sebaceous accumulations, with flat epitheliomatous development in a small colorless mole which had been present for years. Caustic potash treatment was given.

CASE 6.—Mr. —, aged 62. The prepuce was long and had never been retracted. There was a fungous epithelioma, cauliflower like, of the glans. Urine was passed through a crevice in the side of the mass. The inguinal glands were small, hard and hyperplastic. The patient refused to permit their removal. The penis was amputated; a one-inch stump was left. The man was well and at work at last report.

CASE 7.—Mrs. W., aged 40, had a discoid epithelioma on right side of tongue from tooth erosion, constantly kept up. It was thoroughly destroyed with Paquelin cautery. She was well at last report, three or four years later.

CASE 8.—Mr. S., aged 42. The jolt of a car caused him to bite the right side of an already tender tongue. In three months there was extensive involvement of side of tongue and glands of neck. The tongue lesion was not benefited by X-rays. The neck disease was benefited, but the patient died three months later of exhaustion and a hemorrhage from neck.

CASE 9.—Mr. G., aged 52, several months before consultation pinched his lip with pipe-stem. A pinhead lesion followed, which the patient often picked off. Each time it returned larger, finally ulcerating and became a typical epithelioma of the right half of the lower lip on the carmine border. There was early involvement of submaxillary glands. The case was aggravated by the patient's use of some caustic paste. When first seen over half of the lower lip down to the gum was destroyed; there was involvement of the right oral angle and massive metastasis to lymph glands. From the first he refused all operative treatment. He died in about twelve months from beginning of disease.

CASE 10.—A married woman, under 40, three months pregnant. There was a papillomatous cyst of right breast, becoming cancerous. The breast was amputated. Recovery.

CASE 11.—An old woman was seen in consultation. She had incurable carcinoma of the left zygo-malar-maxillary region which had developed in a sebaceous cyst of many years' standing.

CASE 12.—Mr. C., aged 69, had sarcoma of right shoulder girdle, which had developed three months previous in a dark mole over the right scapular spine. It grew rapidly in three or four weeks to the size of a small orange. It was excised by his attending physician, but recurred, and gradually involved the whole shoulder and the axilla. The patient died within six months.—*The Journal of the American Medical Association, July 7, 1906, Vol. 47, pp. 15-17.*

---

### SODIUM CITRATE IN INFANT FEEDING.*

The infant deprived of the breast must, in relation to his feeding, be regarded as a pathologic problem. That this statement is axiomatic is shown by the fact that more earnest work has been bestowed on infant feeding during the past twenty years than on all other pediatric subjects combined. That the problem is still unsolved the high relative mortality and later morbidity of artificially fed infants bear constant witness.

The six essentials of Cheadle, enunciated by that observer some time in the 80's, bear witness that the essential requirements of an artificial food for babies were broadly understood twenty years ago. Quoting from memory, they are briefly as follows: 1. Sterility. 2. Antiscorbutic property. 3. Quantity. 4. Some animal constituent. 5. Form suitable. 6. Constituency. Much has been learned since Cheadle by amassing clinical results and observations. Meanwhile the deductions from *a priori* reasoning have proven unsatisfactory for the simple reason that so little was known of the physiology of infant digestion. Latterly the premises from which we reasoned have been proven, almost without exception, to be incorrect.

Thus the great American experiment known as the exact percentage method of feeding required that the baby's diet should consist wholly of cow products and water, so modified in its five gross constituents as to coincide quantitatively with similarly named constituents of average breast milk. One other ingredient only was allowable in this feeding mixture, viz., some alkalin solution, as lime water, sodium bicarbonate or magnesia. This dogma, which in connection with laboratories established throughout the country had almost become a cult

---

*Read by A. C. Cotton, M. D., Chicago in the Section on Diseases of Children of the American Medical Association, at the Fifty-seventh Annual Session, June, 1906.

infant feeding, was practically based on seven assumptions, which have recently been proven erroneous, to wit:

1. The qualitative similarity in breast and cow's milk of the lactose, fats, albuminoids, proteids and their chemical combination with the milk salts.

2. The claimed alkalinity of breast milk in contradistinction to the acidity of cow's milk.

3. The possibility of a synthetical substitute for breast milk from cow products alone.

4. The claim that cereals were not allowable because indigestible in the infant's secretions and that they induced dyspepsia and intoxication by acting as decomposing foreign matter.

5. That cereal gruels were no more efficient in the prevention of dense milk coagula than dilution with plain water.

6. That HCl played no part in the digestive process until later infancy.

7. That the important proteid content of the baby's food could be made up from the non-coagulable whey albumin of cow's milk.

The elucidation of facts in refutation of these premises is due to the earnest work of a number of men, prominent among whom as members of this association may be mentioned Chapin on cereal gruels and gastric development, Kerley on milk reaction and alkalinization, Shaw on HCl secretion in infants, Stern on milk fats, Southworth on the art versus the science of infant feeding, Morse on acidified milk and buttermilk feeding, and the recent unsurpassed work in chemistry of milk by Van Slyke and Hart at Geneva. Most of these contributions have enriched our literature and become familiar to all. The subject is still a broad one. I disclaim any intention of attempting in this limited paper to deal with it *in toto* and indulge in these preliminary observations merely to emphasize the fact that no royal road to successful feeding by exact mathematical formulas has yet been found. Nor do I wish to convey the impression that I would belittle the value of attempts at accuracy in determining the component parts of the infant's dietary.

Great credit is due and will ever be associated with the name of Dr. Rotch as the founder of a system which has not only served as an hypothesis for tentative feeding, but has stimulated to greater accuracy and more careful clinical observation and recording of results than was possible by any other method. As an enthusiastic advocate of the so-called American method, I am on record frequently both at home and abroad. I may be pardoned if I suggest that the tendency in general is too much along the line of the refinement of mathematical formulas to the neglect of the obvious importance of gastric development along normal physiologic lines.

The spectacle of a marantic infant with persistent curds in the stools, though cow proteids have been reduced to the extreme limit of attenuation by dilution with water, is a familiar one. So, also, too commonly in evidence is the child fed long, but not nourished, on whey proteids and cream whose gastric vigor has not progressed beyond that of early infancy.

Important as the role of carbohydrates and fats in infant development may be and difficult as the problem of properly dealing with fats is, it is to the management of the proteids that I beg to call your attention in the few minutes allowed. Their importance in the nutrition of the child needs no emphasis, as it is universally accepted both theoretically and clinically.

The intolerance of the infant's digestive organs to cow proteids, so widely different from those for which they were intended, is also well known. Of the many methods in vogue for the establishment of toleration of these refractory curds the one most common, unfortunately, is their quantitative reduction by dilution far below percentages absolutely necessary for the development and growth of the child. In fact, the common advice to infant feeders on percentages of milk in recognition of its refractoriness is either to restrict the proteid percentage far below that which is known to obtain in average breast milk (as though Nature would commit the absurdity of elaborating the necessary amount of proteid from even a less quantity when pre ented in the more obstinate form of cow casein), or to substitute the non-coagulable proteids of whey.

In either case Nature is cheated of her absolute demands. The pathetic malnutritional results of low proteid feeding, both immediate and remote, are too familiar to us all to need further elaboration. Aside from this slow starvation and stunted growth with its diminished resistance to intercurrent disease from insufficiency of nitrogenous food, abundance of proteids is demanded in a coagulable form for the normal development of the gastric function, as ably shown by Chapin and corroborated by a host of clinical observers. This the infant gets at the mother's breast in proteids which clot on admixture with the gastric contents in soft, flocculent, semi-solid coagula which readily pass the pylorus in a form suitable to intestinal digestion and absorption.

The important question, then, in feeding cow's milk is not how to reduce the proteids and sustain life, but how to *increase* the proteids and maintain unimpaired digestion. Buttermilk has been tried; acidulated milk has been tried; admixture with gruels has been tried; koumiss, matzoon and kephir milk have been tried; the addition of various alkalies is much in vogue; all with varying degrees of success, and each measure has its ardent advocates. That the question is not yet settled this diversity of opinions amply indicates. It is still an open field, and the cry that no chemical tampering with the milk should be encouraged need deter no one, since it is proven beyond dispute that cow's milk, however modified, is not a natural food for the human infant. The more orthodox observers of the original laboratory percentage modification have from the first chemically tampered with the milk in the addition of lime water, sodium bicarbonate, etc., in the mistaken notion of humanizing the mixture by rendering it alkalin. That we reached further than we intended in our administration of alkalies and secured toleration of the curds through neutralization of the normal gastric acids does not lessen the evils of inhibition of those digestive processes which can occur only in an acid medium.

Since deductive methods from *a priori* reasoning have thus far failed of a satisfactory solution of this problem, let us welcome inductive methods conducted along rational lines, since massed clinical evidence must ever be the tribunal before which all methods must come to trial. Whether *secundus artem* or *secundus scientiam*, it is the greatest number of babies who live and thrive and resist disease that demonstrates the merits of their hygiene.

### AUTHOR'S EXPERIENCE.

It is with the firm conviction that sodium citrate, through its inhibition of dense coagulation of cow's milk in the presence of an acid and rennin, may prove valuable in the solution of the proteid problem that I present a brief resume of my experience with this agent.

More than two years ago in a personal letter from one of my assistants, Dr. J. W. Vanderslice, who was studying abroad, my attention was called to this use of sodium citrate. The reports from Dr. F. J. Poynton's clinic at Great Ormond Street, London, from which the writer as an eye-witness drew his conclusions, seemed sufficient to justify a careful consideration of this new method of overcoming some of the obstacles in the adaptation of cow's proteids to infant digestion.

Rather cautiously, I began prescribing the citrate in cases in which varying milk mixtures had been used with poor success. Later, as I found that infants would tolerate a larger proportion of the milk in the feeding mixture when citrated than by any other modification known to me, I used it more boldly, so that during the past two years I find a record of its employment for a longer or shorter period in more than fifty cases in both hospital and private practice. I have here, by the courtesy of Dr. J. D. Merrill, a report of eight cases in which she has carefully observed its effects for a considerable time, also by the courtesy of Dr. Vanderslice a history of twenty-nine cases reported by him at different times to the Chicago Pediatric Society. In addition to this I have read carefully Dr. Shaw's report of twenty-two cases, making a total of one hundred and twelve cases, embracing nearly all conditions from simple dyspepsia to marasmus and ranging in age from the new-born to adults who have suffered from milk dyspepsia.

Sodium citrate being very soluble in water, the method of employment is simple, as follows: An aqueous solution is ordered containing from one to five grains to the dram. A quantity of this solution is furnished the mother or nurse with instructions to add to the baby's bottle immediately before feeding enough of the solution to represent one, two or even three grains of the citrate to each ounce of milk in the feeding mixture, according to the prescriber's idea of the requirements. The feeding mixture may consist of varying dilutions of milk with water or gruel with the addition of cane or milk sugar, with or without cream. No alkalies are added, the sodium citrate used being a neutral salt. A most noticeable feature in this method of feeding is the large proportion of milk in the feeding mixture that the infant will tolerate without evidence of gastric disturbance or the appearance of any considerable amount of undigested casein in the stools. In fact, the stools of babies fed on citrated milk have come to be regarded by Drs. Merrill, Vanderslice and myself as positively characteristic, being firm enough to show form on the diaper, free from fecal odor and homogeneous in color and consistency. The slight tendency to constipation mentioned by Dr. Vanderslice I have observed in a number of cases in which I attributed it to the low percentage of fat in the food, consisting of equal parts of milk and water or even one part of milk to two of water. In but few instances have I observed this constipation where the fat contents of the mixture equaled 3 per cent.

One indication for the increase of the sodium citrate, even in some cases to as high as three grains to the ounce of milk, is vomiting of curds. Another indication is the appearance of curds in the stools, care being taken to exclude indigestion from excess or intolerance of fats. Of the stools, J. H. Salisbury, professor of chemistry, to whom they were submitted, reports as follows:

"Three samples of the feces of infants fed with milk modified by the addition of citrate of sodium were examined. The feces were yellow, of a moderately firm consistence and remarkably homogeneous. On microscopic examination a few round masses, probably calcium soaps, could be seen scattered through the otherwise very fine debris. In two specimens short crystals of soaps could be detected. No free fat was found. On treatment with acetic acid more or less acid crystals could be observed and in one specimen this was especially marked. Chemical examination of one specimen for proteids was negative."

The duration of the administration of the sodium citrate, as well as the quantity employed, varies considerably in different cases, the purpose being to bring the baby's feeding up toward whole milk as rapidly as possible. As toleration is established the amount of citrate is reduced to one, to one-half and to one-fourth of a grain per ounce of milk until it can be discontinued. It happens not infrequently that the citrate is profitably resumed on the recurrence of signs of indigestion. In but six cases have I felt obliged to discontinue the citrated milk and adopt other methods of feeding. Some of these were among my early cases which, if occurring later, would possibly have received a more persistent treatment with citrated milk.

In consideration of this subject three questions naturally arise: 1. Is the employment of sodium citrate any better than other methods of rendering cow proteids tolerable and digestible? 2. Is its employment harmful? 3. In what manner does it act?

In answer to the first I would say that this method seems to allow a more rapid increase in the proportion of milk ingested than any other known to me. Moreover, the frequency of relief from milk indigestion on the addition of the citrate and the favorable reports from all whom I have known to give it a thorough trial are not without significance. In regard to its harmfulness no case of citrated milk feeding has been brought to my attention in which there appeared to be cause for regret because of the employment of this method.

Concerning its manner of action in a chemical sense there appears to be some difference of opinion. Professor Salisbury, above quoted, to whom the question was submitted, states as follows:

"Citrate of calcium is insoluble in water, but dissolves readily in solutions of the alkali citrates. It is to be presumed, therefore, that when a solution of sodium citrate is added to milk which contains calcium in combination with casein a reaction would occur producing a sodium combination of casein and an insoluble calcium citrate. The latter would dissolve on the addition of an access of sodium citrate. It is to be presumed, therefore, that the milk treated with citrate of sodium contains a sodium compound of casein and calcium citrate held in solution by the presence of sodium citrate. Such milk would not yield a curd containing an excess of calcium, but the calcium would be found in the whey as well as in the curd. The experiments of Dr. R. Aibinder do not contradict the theory of Poynton that sodium citrate acts by separating the calcium from the casein, thus rendering it less easily coagulable. Sodium citrate would react with hydrochloric acid solution to form sodium chlorid and citric acid. The decomposition would not be complete but so nearly so that a large quantity of sodium citrate would neutralize nearly all the free hydrochloric acid of the gastric juice. Too large an amount of sodium citrate in the milk might, therefore, interfere with the digestion of proteids in the stomach. Such digestion might occur in the intestine from the action of the pancreatic juice."

The physical behavior of citrated milk *in vitro* is obvious and may be demonstrated in a simple manner suggested by Wright and Poynton, who first exploited this method of feeding. Into each of two test tubes containing equal quantities of milk (to one of which sodium citrate has been added) drop a given quantity of liquid rennet and dilute HCl. In both milks coagulation occurs; with this difference, that the citrated milk clots less promptly and the resultant curd is softer, less dense, more nearly resembling the curd of breast milk.

### DR. ALLEN'S EXPERIMENTS.

My assistant, Dr. F. W. Allin, as the result of more than a hundred careful comparisons, has obtained the following results:

*Materials Used.*—Ordinary dairy milk was used in these experiments. Wyeth's liquid rennet was used for curdling agent, which was always added last. A 1 per cent. hydrochloric acid solution and 4 per cent. sodium citrate solution was used. Two drops of HCl in 5 c.c. milk equals .025 per cent. Five drops of sodium citrate solution equals .25 per cent. One grain of sodium citrate to the ounce of milk would be .20 per cent. The gruels were standardized to one ounce of flour or oatmeal to the quart of water and cooked three hours in a double boiler.

*Conditions.*—The experiments were all performed at 40 C. in glass test tubes. The milk was used as whole milk or diluted with water, flour gruel or oatmeal gruel. The dilutions made were two-thirds, one-half, one-third, one-fourth, one-eighth milk.

*Results.*—All experiments were repeated many times. The accompanying summary is a tabulation of results. The time recorded is the average of the results.

### CONCLUSIONS.

1. Sodium citrate in .25 per cent. or more retards, and very high percentages will inhibit coagulation.
2. The presence of HCl hastens coagulation.
3. Diluting milk generally retards coagulation.
4. Gruels appear to have little or no effect in retarding coagulation more than water when the citrate is used.
5. The coagula of citrated milk are softer, smoother and more jelly-like or more flocculent than those of milk not thus treated.

### EXPERIMENTS WITH CITRATED MILK.

### WHOLE MILK.

| Milk. c.c. | Liquid Rennet. m | HCl. 1% m. | Sod. Cit. m. | Time. min. | Curd. |
|---|---|---|---|---|---|
| 5 | 5 | 2 | 0 | 3 | Solid, firm. |
| 5 | 5 | 2 | 5 | 9 | Solid, soft. |
| 5 | 5 | 0 | 0 | 5 | Solid, firm. |
| 5 | 5 | 0 | 5 | 15 | Solid, soft. |
| 5 | 5 | 2 | 1 | 4 | Solid, firm. |
| 5 | 5 | 2 | 2 | 4½ | Solid, firm. |
| 5 | 5 | 2 | 3 | 5½ | Solid, firm. |
| 5 | 5 | 2 | 4 | 7 | Solid, softer. |
| 5 | 5 | 2 | 10 | 12 | Flocculi, no curd. |
| 5 | 5 | 10 | 0 | 1 | Solid, hard. |
| 5 | 0 | 20 | 0 | ...... | No curd. |
| 5 | 0 | 20 | 5 | ...... | No curd. |
| | | HCl 5% | | | |
| 5 | 0 | 1 | 0 | 1 hr. | 0 |
| 5 | 0 | 2 | 0 | " | 0 |
| 5 | 0 | 3 | 0 | " | 0 |
| 5 | 0 | 4 | 0 | " | 0 |
| 5 | 0 | 5 | 0 | 15 | Small flocculi. |
| 5 | 0 | 10 | 0 | 15 | Few flocculi. |

### DILUTED MILK

| Milk. | Liquid Rennet. | HCl. 1% | Sod Cit. | Dilu-ent. | Time. | Curd. |
|---|---|---|---|---|---|---|
| ⅔ | 5 | 2 | 0 | Water | 3 | Solid, firm. |
| ⅔ | 5 | 2 | 5 | Water | 5 | Solid. |
| ⅔ | 5 | 2 | 0 | Gruel | 3 | Solid, firm. |
| ⅔ | 5 | 2 | 5 | Gruel | 20 | No change |
| ½ | 5 | 2 | 0 | Water | 3 | Solid or co'rse granular. |
| ½ | 5 | 2 | 5 | Water | 7 | Solid |
| ½ | 5 | 2 | 0 | Gruel | 3 | Solid, firm. |
| ½ | 5 | 2 | 5 | Gruel | 20 | No change. |
| ½ | 5 | 2 | 2½ c c. | .... | 0 | No change. |
| ½ | 5 | 0 | 0 | Water | 12 | Soft, solid. |
| ⅓ | 5 | 1 | 0 | Water | 5 | Granular. |
| ¼ | 5 | 2 | 0 | Water | 5 | Hard, granular. |
| ¼ | 5 | 2 | 5 | Water | 5 | No change. |
| ¼ | 5 | 2 | 0 | Gruel | 4 | Granular or solid. |
| ¼ | 5 | 2 | 5 | Gruel | 0 | No change. |
| ⅛ | 5 | 2 | 0 | Water | 6 | Small solid. |
| ⅛ | 5 | 2 | 5 | Water | 0 | No change. |

The simplicity of this method commends it, especially in dispensary and out-patient practice where the mother's demand for "medicine" for the baby's dyspepsia may be met by the standard solution of sodium citrate to be administered in teaspoonful dose in each bottle of the feeding mixture. In private practice it furnishes another rational method of infant feeding.—*Journal A. M. A., Oct. 6, 1906.*

---

### A REPORT OF THE MANAGEMENT OF TUBERCULAR PATIENTS APPLYING FOR TREATMENT TO THE DISPENSARY OF THE SAMARITAN HOSPITAL, PHILADELPHIA.

BY MERVIN ROSS TAYLOR, M.D.,

Chief of the Medical Clinic and Adjunct Professor of Materia Medica at the Medical Department, Temple College, Philadelphia.

In considering the work done respecting the management of tubercular patients applying to the Samaritan Hospital dispensary for treatment, I may say that since my appointment four years ago there have been forty-two patients who have suffered from pulmonary tuberculosis. Of these forty-two, eighteen cases were classified as having the disease in its advanced stage, namely, showing well-marked areas of consolidation with cavity formation and constitutional disturbances. Seventeen cases were classified as moderately advanced, showing well-marked changes on percussion and auscultation, with more or less constitutional disturbance. Seven cases were classified as in the incipient stage, or showing but slight physical signs confined to one or both apices, and with mild constitutional symptoms.

Of the occupations showing the greater number of victims, those unfortunate beings working in mills and factories, or, in other words, those engaged in indoor work, give us the high average of twenty-six cases out of our total of forty-two.

It is also of interest to note that two cases of the disease occurred in Italian street-sweepers, and that thirty-three occurred in males and nine in females.

Now as the management of pulmonary tuberculosis has within the last decade and a half become more or less revolutionized from a drug-administering disease to one based on sound dietetic, hygienic, and prophylactic principles for its cure and prevention, I believe that in general our aim and object should be:

First to so instruct our patients in the execution of exact preventive measures necessary for them to take in order to lessen the spread of the disease, or, in other words, lift the veil of ignorance which still abounds in respect to its transmission from one individual to another.

Second, to insure a maximum of rest and fresh air.

Third, that milk and raw eggs, if tolerated, form the basis of diet.

Fourth, that a resort to drugs be made only when necessity arises.

In order to follow out the first rule bearing on prophylaxis and the spread of the disease, I had these rules printed, which are a modification of the rules adopted by the Flick Institute of this city, for distribution to each patient applying for treatment:

1. Don't spit on the pavement, on the street, nor into any place where you can not destroy the germs which you spit up.

2. Do not swallow any spit which comes up from your lungs or which comes out of the back part of your throat, as it exposes you to complications.

3. Spit into a spit-cup when it is possible to do so.

4. Always use a spit-cup with a handle to it, so that you can hold it close to your mouth.

5. When you use a china or earthenware spit-cup, always keep chlorinated lime and water in it, and scald out the spit-cup once or twice a day with boiling water.

6. When you use a tin spit-cup with a paper spit-cup inside, burn the paper cup at least once a day and scald the tin cup with boiling water.

7. Never use a handkerchief or a rag or any material other than paper to spit in or to wipe your mouth with unless it can be immediately burned after using.

8. When you can not spit into a spit-cup, spit into a paper napkin.

9. Always use a paper napkin to wipe your mouth with, after spitting, and be careful not to soil your hands.

10. Always carry a cheap paper bag in your pocket or caba to put paper napkins in which you have used.

11. When you have used a paper napkin, either to spit in or to wipe your mouth with, fold it up carefully and put it away in a paper bag.

12. Every evening, before going to bed, burn your paper bag together with the napkins you have deposited in it.

13. Do not let any spit get on your clothing, or your lips and hands, or your bedclothes or carpets or furniture, or on anything about you, wherever you may be.

14. If, by any accident, any spit should be deposited anywhere else than in your spit-cup or in your paper napkin, take pains at once to destroy it, either by taking it up and putting it in the fire or by putting chlorinated lime and water on it.

15. If you have a mustache or beard, shave it off or crop it close.

16. Always wash your lips and hands before eating or drinking, and rinse out your mouth.

17. If you have a running sore, take up the matter which is given off with absorbent cotton and burn it.

18. Don't blow your breath on to hot milk or any other food substance in order to cool it before giving it to others to take.

19. Avoid handshaking and kissing. These customs are dangerous to you as well as to others. They may give *others* consumption; they may bring *you* colds and influenzas which will greatly aggravate your disease and may prevent your recovery.

20. Do not cough if you can help it. You can control your cough to a great extent by will power. When you cough severely hold a paper napkin to your mouth so as not to throw out spit while coughing.

21. Sit outdoors all you can. If you have no other place to sit than the pavement, sit on the pavement in front of your house.

22. Don't take any exercise except upon the advice of your doctor.

23. Always sleep with your windows open, no difference what the weather may be.

24. Avoid fatigue. One single fatigue may change the course of your disease from a favorable one to an unfavorable one.

25. Go to bed early. If you are working, lie down when you have a few minutes to spare.

26. Don't take any medicine unless it has been prescribed by your physician. Medicine may do you harm as well as good.

27. Don't use alcoholic stimulants of any kind.

28. Don't eat pastries or dainties. They do not nourish you and they may upset your stomach.

29. Take your milk and raw eggs whether you like it or not.

30. Keep up your courage. Make a brave fight for your life. Do what you are told to do as though your recovery depended upon the carrying out of every little detail.

31. Always keep in mind that consumption can be cured in many cases and that it can be prevented in all cases.

32. If your own disease is too far advanced for you to recover, console yourself with the idea that you can keep those who are near and dear to you from getting it.

Patients are earnestly requested to read these over at least once a week, and adhere to them, and tack them up in a conspicuous place in their bedrooms.

By this means of placing within their hands set instructions for them to follow rather than giving them mere verbal directions as to the care of sputum, etc., I felt that I was best able to elevate my patients up to a higher standard of moral responsibility in carrying out protective measures which they owe not only to themselves and their families, but to the community at large.

The securing of a maximum amount of physical rest in the average ambulatory case of tuberculosis applying to a hospital for treatment is, I think, one of the most difficult problems that the dispensary physician is called upon to solve, for the reason that the vast majority of our cases occur in the laboring classes, who are solely dependent upon their meager daily earnings to sustain perhaps not only themselves but their families.

However, it is my custom to advise, whenever I can, the giving up of all work for as long a time as possible, with the following instructions as to rest and fresh air:

Patients should retire for the night at approximately nine o'clock, and not rise until nine o'clock the next morning. This gives them twelve hours of perfect rest in bed. They are told to sleep with all the windows open; to provide themselves with plenty of bedclothes, as chilling of the body by draughts is not only improper but dangerous; and a small, closely-fitting skull cap, or, better, a knitted hood, for the head should be worn in severe weather.

They should sit out in the open air, warmly clad as much as possible during the day, taking a moderate amount of gentle exercise, always avoiding fatigue.

The main contraindications to exercise are hemoptysis and pyrexia. Whenever blood-spitting exists, even though it be extremely slight, I direct that the patient return at once to his home and remain quietly in bed for at least three days, or longer if the sputum be still blood-stained. Of the drugs which are claimed to exert a hemostatic effect in this condition, adrenalin chloride has, in my experience, yielded the best results, although it is not, I think, generally used.

I order that the patient shall take 10 drops of a 1 to 2000 solution every hour for three or four hours, then the same amount every two hours, gradually cutting the dose down as the symptom disappears, always instructing the patient to seek outside medical aid should the hemorrhage become worse. After the three or four days' rest in bed, and if able, they are told to return to the dispensary for examination and further advice.

The epidemicity of hemorrhage in tuberculosis has been noted by careful observers for many years, causing us to look upon it merely as a curiosity, but no attempt has been made to give us a proper explanation of it until recently. Flick, observing that because in pneumonia there usually is some evidence of hemorrhage in the rusty sputum, and with the view of throwing light upon the subject, made a series of bacteriological examinations, with the result that such blood-stained sputum revealed numerous pneumococci present, and he further reports an epidemic of hemotysis occurring in his tubercular wards due to the presence of a case of pneumonia having been accidentally admitted.

In support of this statement I have frequently observed at post-mortems that in those cases in which hemorrhage had occurred, either as a terminal event or when it has been more or less marked during the course of the disease, the pneumonic areas which are so often found surrounding the active tubercular lesions were much more marked in these cases than in those which died without having preexisting blood-spitting. Therefore the presumption is that pneumococci would have been found in the sputum of these patients if they had been looked for at the time of hemorrhage, and perhaps previous to its onset.

Accepting then the new etiological significance of epidemic hemorrhage being due, in a large majority of cases, to an engrafted pneumococcic infection, the doubt arises, even knowing this fact, whether or not our present methods of arresting the condition can be improved upon except from a preventive standpoint.

From the first patients are instructed to take raw eggs and milk; my custom being to start on three eggs a day, which are to be beaten up in one quart of milk, and increased one egg and a half-pint of milk every third day, watching closely my patient's digestive functions, and stopping the milk and eggs entirely for a day or two, or cutting the quantity down, upon appearance of the slightest digestive disorder, believing that any substance, be it food or medicine, which when administered disturbs the stomach to such an extent as to produce an absolute intolerance cuts off our main pathway by which foodstuffs gain entrance into the body to nourish it, and so combat the ravages of this an exhausting disease.

It is further my custom to flavor the milk with one of the vegetable flavoring agents, as vanilla or nutmeg, etc. This removes the flat taste of the milk, making it more agreeable to the taste.

In those who have an absolute abhorrence for the milk-and-egg diet, small doses of pure olive oil taken after meals and slowly increased have, in a few cases, proved of value, but at no time have I obtained such results as when the milk-and-egg diet was faithfully taken.

One case was able to take as much as one pint of pure olive oil a day, and showed a gain of fourteen pounds in weight in six weeks. I weigh each patient once a week, or rather direct that he get weighed.

Of the results obtained from the treatment mentioned, I take pleasure in citing the case of a girl:

R. T., aged twenty-one; occupation, that of dressmaker; living at home. She applied for treatment at the dispensary in July, 1904. Diagnosis: Incipient pulmonary tuberculosis.

There was well-marked disseminated consolidation in the apex of the right lung, but without cavity formation. On auscultation over the apex of the left lung there was revealed a slight prolongation of expiration, with a decidedly harsh vesicular murmur. Coughing was severe, with moderate expectoration. Her temperature was 100 degrees, and pulse 95. Night sweats were not severe, but exhaustion, which was her main complaint, was unusually marked, and there were found tubercle bacilli in large quantities in her sputum.

She was at once ordered home, and to remain in bed for one week, at the end of which time her temperature was found to have dropped to normal, and remained so afterward. She followed out religiously the already described form of treatment, with the result that within six months she had gained 42 pounds in weight, and was then taking 12 eggs and 2½ quarts of milk a day.

Last September, when I saw her last, she stated that by necessity she was required to return to her work, and was feeling in good health. She was coughing very little, and her weight was 140 pounds.

Examination of her chest revealed very little evidence of her former trouble, although there was still some prolongation of expiration over both apices, with a harsh vesicular murmur.

This girl, it must be said, lived up to the treatment better than the average patient can be made to do. She occupied a third-story bedroom, slept by herself, practically in the open air, as the room had three large windows, all of which remained open the year round. She gave up her occupation for one year, took her milk and eggs regularly, and, in short, made the best of a brave fight for health.

This is only one of three cases which I have been able to follow upwards of two years, and which have yielded almost similarly gratifying results.

Of the special symptoms which at times demand treatment, that of excessive coughing warrants consideration, especially if the cough be of such severity as to disturb the patients' rest at night. It is my custom to administer heroin hydrochlorate, grain 1-24, every two hours, as it is my opinion that loss of sleep night after night, caused by a troublesome cough, is more detrimental to the health of the individual than any harm which may be produced by a sedative drug of the nature of heroin given in small doses.

For the relief of constipation, which is so often induced by the milk diet, I have found that the aromatic fluid extract of cascara sagrada, taken in small doses on retiring, or both night and morning if necessary, acts very well.

In regard to exercise and the practice of deep breathing, I firmly believe that they are potent for much good, especially the use of those exercises which have for their object the development of the chest and external muscles of respiration. Chest exercise is particularly indicated for those styled the pretubercular individuals, or, in other words, those whom we believe have a marked susceptibility to the disease by reason of their poorly developed chests in presenting marked supraclavicular and infraclavicular depressions, and other evidences which denote defective lung expansion.

Now in conclusion let us hope that before long our city health and charity departments, as well as our general hospitals, will be provided with sufficient funds to give the thousands of consumptive poor of this city sufficient facilities to make use of God's fresh air by day and by night, with other ample facilities for curing the curable and taking care of the hapless tubercular poor. We will then make greater strides toward the solution of the tuberculosis problem, and so realize again the truth in the statement of Pasteur, that it is within the power of man to rid the world of this disease.—*Therapeutic Gazette*, *September, 1906.*

---

### THE TREATMENT OF SYPHILIS.

Some one has said, and with a great deal of truth, that he who understands syphilis in all of its phases has mastered the major part of diagnosis and has solved, to a great extent, the knottiest problems of medical practice. It is consequently a matter of no little importance when we are able to bring together the recent opinions of any considerable number of competent men on any phase of this widespread disease. *The Therapeutic Gazette* for August 15, contains five articles on the Treatment of Syphilis, one, which is especially noteworthy, being contributed by Dr. George Henry Fox, of New York College of Physicians and Surgeons, and the other four coming from the pens of Drs. M. B. Hartzell, of the University of Pennsylvania; W. A. Hardaway, of the Washington University, of St. Louis; Edward Martin, of the University of Pennsylvania and Granville McGowan, of Los Angeles.

These papers are particularly interesting since they indicate very plainly those points upon which there is unison of opinion and those upon which modern students and writers markedly differ, while Dr. Fox's paper points out, with a clearness which we have not seen before, that there is such a thing as "pushing treatment" in syphilis to a point which not only is productive of no good but which, at times, proves actually injurious to the patient. We will consider first the ideas suggested by Dr. Fox and, in considering these ideas, we will bear in mind that the writer is one whose conclusions may be accepted as more than ordinarily authoritative.

We may summarize Dr. Fox's conclusions briefly as follows:

1. One of the commonest errors in our entire therapeutics is the *over-treatment of syphilis.* The treatment is often far worse and causes the patient more discomfort than the disease. It is a distinct misfortune that we place such implicit confidence in pharmacopœial drugs in the treatment of this disease.

2. Syphilis is a chronic exanthematous disease and, like the acute exanthematous diseases (scarlet fever, measles, etc.), tends to self-limitation and may result in complete recovery without the use of any drugs.

3. If physicians would devote their attention to the improvement of the general condition of the patient, to diet, exercise, etc., more brilliant results would occur and less unfortunate sequelae. It is the *vis medicatrix naturae* which is the most important element in the cure of syphilis as well as any other disease.

4. In many obstinate cases where large doses of antisyphilitics do not cause improvement, "pushing the treatment" does no good and may do injury. Of such cases, while increasing doses may benefit one, they will injure twenty. If half the money spent for iodides for such patients were spent for milk and eggs and to provide outdoor life, there would be more and quicker cures. It would be instructive if physicians could see the results of faith cure in this class of cases.

5. While antisyphilitics have a definite curative effect on lesions of syphilis, they will not cure the results of syphilis. Hence the absurdity of vigorously dosing with mercury and iodides every patient who presents deformities or other evidence of past syphilitic infection. It must not be accepted without question that every spot or lesion on one who has had syphilis is syphilitic in character. Aside from the waste of good medicine in such cases, the treatment will often injure the patient who does not need it.

6. The claim that virulent tertiary stages are apt to follow when there is not vigorous treatment in the early stages is not borne out in fact. While this is not proven, it is certain, however, that virulent tertiary stages may follow in spite of early vigorous treatment. There is reason to believe that the virulent pox of years ago was due to the over-vigorous treatment of that day and that the disease has decreased in virulence because the treatment has become less vigorous.

7. The rule of treating in a routine way for two, three and a half or four years is illogical and often harmful. No definite time of treatment can be set. We do not specify that we shall treat scarlet fever for one, three or five weeks, nor can we limit or specify our time of treatment in syphilis.

The foregoing suggestions we have regarded sufficiently important to quote at length and their observance by the medical profession will doubtless result in better results and more contented patients.

The four other writers deal with other subjects in which we will note considerable variance of opinion. For convenience of comparison our abstracts are compiled for the group and note for the individual papers.

1. *When should treatment begin?*—Hartzell, Hardaway and MacGowan agree that it is hardly safe to begin treatment until the appearance of constitutional symptoms and Hardaway adds that "violation of this cardinal rule leads to an infinite amount of mischief." Martin holds that, when there has been a period of incubation of two weeks with appearance of a papule not otherwise accounted for, this lesion should be excised and examined for spirochæta pallida and, in case this is found, treatment should begin at once. He sees no reason for delaying treatment until the appearance of secondaries and calls attention to the fact that some cases, in which diagnosis could be made on the initial lesion, present no constitutional symptoms at all.

2. *What treatment shall we begin with?* Hartzell begins treatment with mercury and chalk in small doses, not over 5 to 8 grains per day, and claims that this form of mercury is far less irritating and fully as effective as the protiodide (or green iodide). Hardaway prefers the protiodide (1-6 to ½ grain t. i. d.) with iron or iron and quinine, holding that this preparation is more effective. Martin begins treatment with inunctions supplemented with vapor baths. In case this is impracticable, he uses the protiodide, mercury and chalk or mercurol, selecting them in the order given. MacGowan does not use inunctions if the patient is able to take mercury by mouth without too great digestive disturbance.

3. *What is the treatment for local lesions?* In the eruption of the secondary stage, Hartzell uses (when anything is necessary) calomel ointment (1 drachm to the ounce) applied daily with gentle friction. For the scaly eruption of the palms and soles, he uses an ointment of ammoniated mercury (40 to 60 grains to the ounce). For shallow erosions of the mouth, tongue or pharynx, he uses acid or mercury. For the tertiary ulcerative lesions, he uses mercurial plaster, after washing away secretions with 1:4,000 bichloride solution. For such lesions he advocates also iodoform. MacGowan treats the initial lesion with gauze compresses saturated in 1 :4,000 bichloride solution. Chancre of the tonsil he treats with acid nitrate followed by a mouth wash of 1 :10,000 bichloride in a saturated solution of sodium chlorate. This applies also to chancre of the gums and tongue. Chancre of the lip is to be treated with the acid nitrate followed by an ointment of colloidal mercury. The pain may be reduced by application of an alcoholic solution of methylene blue. Chancre of the meatus or urethra are treated with bougies of 25 to 50 per cent. calomel ointment. For mucous patches of the buccal cavity, use a 25 per cent application of nitrate of silver followed by a mouth-wash of 1 :4,000 bichloride of mercury in a solution of chlorate of potassium or chlorate of sodium.

4. *When and how shall we give iodides?* Hartzell believes that iodides should be given with the appearance of tertiary symptoms and deplores the common custom of giving large doses. He corroborates the statement of Jonathan Hutchinson that small doses accomplish the same results as large doses. Hartzell can not recommend the mixed treatment or combination of iodides and mercury, nor does he have much faith in the various preparations which have been offered to take the place of the iodide of potassium. Hardaway believes that iodides given in the first stage of the disease will control the arthritic pains and neuralgias. However, he advocates reserving the iodides for the late secondary and tertiary stages of the disease. He thinks that at all times when the iodides are given, mercury should be given also and should be continued after the iodides are stopped. Martin believes that iodides may be begun the latter part of the second year, but should be given sparingly.

5. *How long shall treatment last?* Fox says that this depends upon the case and that much harm is done by following any routine as the patient is often treated after his complete recovery and to his great detriment. Hartzell says that treatment should continue for two or three years: the first year continuously; the second year the treatment should be broken and, if symptoms continue to appear during the second year, the treatment should be continued during the third year. Hardaway believes in constant mercurial treatment for six months, then a rest of six weeks with tonic treatment, then a resumption of treatment which will continue in broken form for about two years, while the patient should take a course of treatment of several weeks duration at least once a year for four years. Martin believes in constant treatment for two years and, after the fourth year and for life, the patient should take spring and fall courses of inunctions each covering six to twelve inunctions.

6. *What is the prevailing opinion of hypodermic injections?* Hartzell holds that hypodermic injections of mercury should be reserved for cases where it is necessary to secure marked mercurial impression in a brief space of time, as in cerebral syphilis. He prefers the soluble forms of mercury. Hardaway holds about the same opinion and adds that the hypodermic may be used when other methods of administration are contraindicated or when they are not sufficiently effective. He prefers the bichloride of mercury for hypodermic use. Martin praises the hypodermic method, but states that it may cause almost unbearable pain against which we have no way of guarding. Mac-

Gowan praises the hypodermic method with the use of the soluble salts, but condemns the use of the insoluble salts.—*The Chicago Clinic and Pure Water Journal*, *Sept.*, *'06.*

---

### THE ACCUMULATIVE EXPERIENCE OF THE PROFESSION IN THE USE OF ROENTGEN RAYS IN THE TREATMENT OF ACNE, ACNE ROSACEA, ECZEMA AND PSORIASIS.*

A. R. Biddle concludes his paper as follows: I feel justified in presenting the following conclusions, which I trust will be ratified or modified to meet the views of the members, that the result may be an authoritative expression of this society:

Time, a decade, through the experience of a large number of competent observers, has given to Roentgen rays a valuable and, by reason of improved methods for the measure of dosage and perfection of machinery and technique, an ever-increasing usefulness as a therapeutic agent in the local treatment of acne, acne rosacea, eczema, and psoriasis. Conservatively used, with due regard to their well-recognized dangers, especially in these non-malignant dermatoses, a knowledge of their efficiency and limitations, and with proper technique, they are useful in selected cases, not as a routine nor as the only treatment, but as an addition to our other remedies, especially in those cases which have resisted ordinary treatment, and in which there is a marked infiltration of the tissues.

*Acne.*—While X-ray therapy is applied by a few to all types of acne, it finds its greatest usefulness in the rebellious, indurated, deep-seated, sluggish, staphylococcic, pustular type, which has resisted all other measures. As a routine treatment the dangers to the face from exposure even with the best technique outweigh its advantages over other tried measures. In every case the exposures should be given with caution; they should be mild and of short duration. The rays seem to exercise their greatest influence in those with coarse seborrheal and pale, pasty skins, with a natural predisposition to acne and comedones.

---

*Read before the Seventh Annual Roentgen Ray Society at Niagara Falls, N. Y., Aug 29 to 31, 1906.

Although the experience of operators seem to vary greatly as to the time necessary to effect a cure, it would seem that the more permanent results are obtained in those cases in which exposures are given in cycles. The preponderance of opinion is to the effect that, though the time for effecting a cure is not materially lessened, cases otherwise incurable are much benefited and relapses are less frequent.

In *acne rosacea* the results are not brilliant. The influence is greatest in those conditions in which the glandular inflammation predominates. The effect on the dilated blood-vessels and upon the tumefaction of the nose is not appreciable. The treatment must be prolonged, though in those cases in which improvement is noticed it seems to be shortened in comparison with other methods. The same caution must be observed as in the cases of acne.

*Eczema.*—The chronic, infiltrated patch, found as a type in eczema of the hands and of the feet, will often yield to X-ray therapy, when it has resisted with a dogged persistence all other remedies. In selected cases of subacute inflammation it may be found useful, but it should never be applied to the acute stages of the disease except in cases of unusually severe and protracted pruritus, to the eczemas of childhood, except in most obstinate cases, or to an eczema otherwise easily managed. Some brilliant results are reported, and it would seem that the duration of treatment is shortened, but recurrences are frequent.

*Psoriasis.*—To the persistent, large, infiltrated plaque, or to the aggregation of smaller lesions, which nothing else seems to affect with any degree of permanency, is radiotherapy applicable with the assurance of good results? The treatment must be prolonged, and relapses are not infrequent. Reports are received of the successful treatment of psoriasis of the nail and of a few cases of generalized psoriasis, but on the whole, except in a few isolated cases, which have yielded quickly and apparently to the influence of the rays, the story is one of repeated disappointment.

The claims of the early enthusiast have not been realized, and such claims seldom are. But in the hands of the competent operator, with the present more accurate means of measuring the dosage, and the gradual improvement in technique, X-ray therapy in general is finding its level, and within its limitations the results are more certain and permanent. Were the dermatologist more expert with the machine and did he give it his personal attention, instead of relying upon the uncertainty of an assistant, and were the radiotherapist a better diagnostician of a dermatological lesion, more uniform results would probably be reported. The causes of failure with the one lies in his ignorance of his agency and his improper technique; with the other, in his inability to recognize the true character of the lesion and its interdependence upon other constitutional conditions.

Ignorance, faulty judgment, and inexperience of the operator (defective technique), overconfidence and rashness or timidity in its use (for I believe with the history of past mishaps as their precedent more men under expose than overexpose), have done much to belittle the value of X-ray therapy. To this may be addedas causes of failure: defect of machinery, uncertainty of reaction in the individual skin, inability of the operator from one cause or another to select cases appropriate for treatment, time and expense necessary to a successful outcome, and uncertainty of results.

The dangers, namely, permanent telangiectasis, warty growths, burns, atrophy or increased and permanent pigmentation of the skin, growth of downy hairs, etc., should always be kept in mind. Fortunately, the burn is of less frequent occurrence, but the dilated blood-vessels and the atrophy may not present themselves for several months after the exposures have been discontinued, even when the exposures have been mild and have produced little or no inflammatory reaction (Hyde and Montgomery). And so we find that X-ray therapy is contraindicated in all types of acute inflammation or during acute exacerbation of a chronic condition, and in childhood, when the skin is too delicate and dangers are too serious, except as a *dernier resort*.

The most successful practitioner will be he who combines an extensive clinical experience with a thorough knowledge of the principles and practice of Roentgenology; he who, never neglecting an opportunity to improve his knowledge of the one, keeps himself abreast the advance of the others.—*Medical Age, Sept. 25, '06.*

---

### TREATMENT OF ECLAMPSIA GRAVIDARUM.

In a practical note on the treatment of puerperal convulsions, Faix and Herbinet briefly review the approved therapeutics of this condition (*La Gazette Medicale du Centre*, through *Le Journal de Medicine*, May 6, 1906). When confronted by a case of pregnancy attacked by convulsions, the first effort should be to strive against the toxic agents and to remove them as rapidly as possible from the system. This indication is admirably met by bleeding. When a vein can not be conveniently opened at the bend of the elbow, we may take blood from the internal saphenous vein in front of the internal malleolus, where it can be easily felt lying on the surface of the bone. From it can readily be obtained from 500 to 800 grammes of blood, according to the corpulency of the patient. Having removed part of the poisoned blood and reduced the arterial tension by the venesection, other means may be added, such as purgatives, enteroclysis, inhalations of oxygen and a water diet. The saline purgatives employed, such as Carlsbad or Seidlitz salt, by causing a flow from the intestine, relieve the organism of toxines and act as derivatives of the intestinal mucosa. Enteroclysis completes the antitoxic treatment. By the aid of a long rectal tube, from 20 to 30 liters of boiled water may be thrown into the bowels; and this may be done twice daily, morning and night. By the mouth, water is also given; either willingly or by force, the patient is made to take 200 grammes of boiled water every hour. If this be done, there is no need to resort to the subcutaneous injections of artificial serum. All the water needed will be taken from the stomach and intestine when given in these large quantities. Moreover, it is questionable if the addition of salt to the blood is an advantage in the condition which is essentially uremic, and where there already is difficulty in getting salts out of the system. The intravenous injection is proscribed by the fact that the blood tension is already high, and to increase the volume of blood might directly lead to rupture of a blood-vessel, possibly in the brain. Chloral and chloroform are also excluded as unnecessary. They are not condemned because they are dangerous, but because they are superfluous; their only object being to reduce the number of paroxysms. They may, however, act as an additional toxic element and increase the difficulties of elimination. One accident attending the convulsion, the biting of the tongue, should be guarded against by holding the lower jaw firmly against the upper during the attack, or by doubling a handkerchief into a compress, 4 to 5 centimetres in size, and placing it between the two maxillæ. As regards the obstetrical treatment, the cases are divided into two classes in accordance with the period of the pregnancy at which they appear. If they occur during the early months, which is rare, they are to be treated as simple cases of uremia, knowing that the fetus will probably be lost and the case thereafter progressively improve. If, however, the convulsions continue in spite of treatment, it will be necessary to empty the uterus. When, as is usually the case, the eclampsia occurs at the time of labor, the dilatation may be incomplete; it is then advisable to wait, no matter what may be the condition of the infant. The viability of the latter is already gravely compromised, and it is not worth while to undertake the risks of a forced dilatation in a uterus which is frequently tetanized. On the contrary, if the dilatation is complete, the delivery should be practiced as quickly as possible, either by forceps or version, according to circumstances. The tissues being edematous, care should be taken to prevent lacerations of the perineum, which are, however, difficult to avoid. When they happen immediate union should be attempted, but a secondary perineorraphy will often be required. An intrauterine injection of boiled water should be given after the delivery. On account of the need of using purgatives and the increased danger of infection, the usual vulvar pad and T

bandage should not be applied. In a case of severe convulsions after the seventh month of pregnancy, if the patient's death should occur while the heart of the fetus can still be detected, the attendant should not lose a moment in performing the Cæsarean operation to save the infant. If the eclamptic attack should occur during childbirth, and both mother and child survive, it will be asked if she may nurse her infant. This is answered in the affirmative, and this practice is followed at the maternity hospital without any bad effects to the child. —*N. Y. and Phil. Medical Journal, October 6, 1906.*

---

### MILK SUPPLY.

Harcourt, among other things, makes note of the following: The taste and purity of milk are influenced in many ways. Bad flavors may be given to it from the food eaten by the cow, by the foul air breathed by the animal, by the products of certain germs, and by the direct absorption of bad odors. Milk is practically never free from bacteria, and even with the utmost care it is often impossible to prevent further contamination. Hairs and scales from the body, dust from the udder, sides of the cow and from the air in the stable, and even dried particles of animal manure all loaded with germ life, find their way into the milk during milking operations. During bottling and delivery there is still further chance for contamination. Freshly drawn milk that has been produced under strictly sanitary methods should contain only a few hundred bacteria to the cubic centimetre. Such milk will remain sweet for a long time, especially if kept at a low temperature. Country milk produced from reasonably clean cows in clean stables, and kept at a low temperature, should not contain more than from 5,000 to 20,000 bacteria per cubic centimetre when first placed on the market. Such milk, if free from bad flavors, is satisfactory for general consumption. A large number of bacteria in the milk indicates that the milk is old, or that it has been kept at too high a temperature, or that it has been produced under unsanitary conditions. If the germs are lactic acid organisms the milk may be old, but not necessarily dirty. The presence of many liquefying organisms is an indication that filth of some sort has gained access to the milk. Other things being equal the price of milk should depend upon its composition: The more fat it contains the higher should be the price. Pasteurization of milk is often necessary in the case of milk handled by large companies, where it is often several days' old before it reaches the consumer. But where it can be bottled immediately after it is drawn, and cooled and delivered direct to the consumer pasteurization is not necessary. The addition of preservatives of any kind should not be countenanced, and every effort made to prevent their use. A really good milk can not be produced as cheaply as the ordinary kind; hence the price is bound to be increased. Glaister sums up the desiderata required of milk as follows: 1. A guarantee at the hands of a qualified sanitary official, after inspection of premises, animals and milking operations of every dairy farm sending milk to a city, that cleanly conditions had been secured, and that clean milk was being supplied. Such a guarantee in the hands of a milk seller would command the confidence of the public and would be calculated to reduce milk borne disease. 2. The systematic official inspection of dairy farms and their water supplies. 3. Registration, after inspection, of such forms, and of places of milk distribution in cities and towns. The observance of such conditions as the foregoing might be tested by the establishment of certain standards, on the part of the sanitary authorities, as follows: (*a*) A standard of cleanliness respecting amount of deposit of foreign matter. (*b*) A bacteriological standard indicating the maximum number of organisms permissible in milk in summer. (*c*) A standard of temperature to ensure the proper cooling of milk, and the maintenance of a low temperature during transit. (*d*) A prohibition of the sale of milk which, within six hours after purchase, could not be boiled without coagulating.—*British Medical Journal*, *September 22, 1906.*

### ROENTGEN THERAPY OF TUBERCULAR GLANDS.

BY G. H. SVOVER, M.D.

After recounting various methods of treating this condition the author considers two methods only to be used—surgical and radiotherapy. Surgery, he says, if not well done, and a complete eradication of the diseased tissues secured, had better be left undone. He considers the X-ray very useful in these cases, and while not claiming it to be a cure for every case, he reports several cases in which it was used with marked success, He has used in these cases artificial fluorescence, as suggested by W. J. Morton, M.D., but seems to have been doubtful of its utility, but employed it to give the patient the benefit of the doubt. The first case reported was in an extremely bad general condition, and was "a whole hospital ward in herself," for the number and variety of complications. She had two tubercular glands on the right side of the neck, one of which had been suppurating for several months. The surgeon stated to him, when sending the patient, that if she lived very long it would be a miracle. The suppuration ceased under the X-ray and the other gland disappeared. The former hopeless invalid is now a self-supporting member of society. The second case reported has had tubercular glands for several years. They have been opened and pus evacuated repeatedly. When she came to him there was an immense mass on each side of the neck, extending from the lobe of the ear down into the shoulder and a mass under the jaw. She was treated steadily for one year, and now, nineteen months after the treatment was completed, an observer, standing a short distance away, would notice nothing abnormal. The third case, a boy, had tubercular glands on each side of the neck. He was a delicate-looking child, whose mother died from tuberculosis. He was treated for five months, at the end of which time no trouble was apparent, and he had gained considerable in weight. Twenty-one months after treatment he is well and a healthy-looking boy. Case number four was a girl of ten years, daughter of a tubercular mother. This patient had a number of tubercular glands on the right side of the neck. On the left were several enlarged glands, one of which was as large as a pigeon's egg. She is still under treatment. The gland on the right side has practically disappeared. The fifth case presented masses of large glands on each side of the neck and under the jaw, under treatment at present; they have greatly reduced in size, and are now represented by hard lumps, composed probably mostly of fibrous tissue. The sixth case had scrofulous glands in both groins, a large and acutely inflamed one in the left axilla, a chain of quite small ones in the right side of neck, a lump the size of an almond in the right breast, and a number of quite large mesenteric glands could be palpated in the abdomen. She had suffered from diarrhea for a long time and had daily rise of temperature and was not a very promising case. Fluorescin was used in conjunction with the X-ray. After fourteen treatments her fever disappeared, and after the nineteenth exposure her diarrhea disappeared. After twenty-one administrations the treatment was discontinued, and after her vacation, when she returned to the office, she was in better health than she had been for many years. The lump in the left breast is hard and only one-fourth its former size, and only traces of the glands in axilla are found. The seventh case has had occasional swellings of the glands of the neck for eight years, which have been gradually increasing in size for the past six years. When she came for treatment, there was a chain of large glands on each side of the neck, and a number under the chin. She received fluorescin in conjunction with the X-ray. After fifteen exposures all the glands in the neck were gone and the others were smaller. After twenty-three exposures treatment was discontinued.—*Denver Medical Journal.*

---

### UTERINE AND OVARIAN PHYSIOLOGY.

Bond, after a series of experiments with rabbits, reported in the *Journal of the American Medical Association*, concludes as follows:

The presence of functionally active ovarian tissue is necessary for the uterine functions, or that portion of it which is concerned with the preparation of the endometrium of a suitable nidus for the imbedding or fertilized ova.

The presence of the uterine or endometrium tissue is not, on the other hand, necessary for the carrying on of ovarian function, either ovulation or the production of the internal secretion associated with estrus.

One function of the endometrium in the anestrous state is the secretion of a saline, watery fluid of low specific gravity, containing a large amount of chloride of sodium solution.

There is some antagonism between this endometric function of saline secretion and that portion of the internal secretion of the ovary which is specially concerned in producing pre-estrous changes in the endometrium preparatory to the imbedding of fertilized ova—that portion of the internal secretion of the ovary which is, in fact, associated with the growth of corpora lutea. Thus the saline uterine secretion is associated with katabolic, the ovarium secretion with anabolic, changes.

The mechanism by means of which the ovary obtains its stimulus from the stimulated endometrium consequent upon the occurrence of pregnancy is a circulatory and not a nervous mechanism. In all probability some substance is manufactured by endometrium or by trophoblast, or by both, which reaches the ovary by way of the blood stream, and is also concerned with the increased activity in the mammary glands.

The bilateral and bicorned uterus in the rabbit may be regarded as one gland so far as the functions of the endometrium is concerned, which is associated with the preparation of suitable nidus for fertilized ova.

The bilateral ovaries may also be regarded as one gland so far as the functions of ovulation and production of internal secretion are concerned.

After removal of one portion or half of this gland, the remaining portion is capable under certain conditions, of undergoing a process of compensatory hypertrophy.

These necessary conditions in the rabbit are (*a*) the presence of the stimulus—namely, copulation—which in this animal normally determines ovulation, or (*b*) the stimulus of pregnancy. Under normal conditions these two stimuli occur together.

These hypertrophic changes in the ovary after unilateral oöphorectomy structurally resemble those changes which normally occur in the ovaries during pregnancy in its early stages.

After unilateral oöphorectomy together with hysterectomy in the rabbit, the hypertrophic stimulus is supplied by repeated copulation.

The prevention by previous hysterectomy of the secretion of the saline fluid by the endometrium of the anestrous uterus favors the overgrowth in the ovary of lutein tissue.

The facts now established are :

The endometrium has a secretion peculiar to the anestrous state.

There is evidence that some substance is elaborated by the pregnant uterus which stimulates the growth of corpora lutea in transplanted ovaries.

Bond declares that we must, therefore, reconsider this matter and suggests that the ovary elaborates only one internal secretion having an influence on the uterus of an anabolic character, which at recurring intervals increases in amount and produces the phenomenon of pro-estrus and estrus.

---

### TRIBUTE TO THE LATE DR. E. D. FERGUSON, OF TROY.

At a special meeting of the board of directors of the Samaritan Hospital, held September 10, 1906, the following minute was unanimously adopted relating to the death of Dr. E. D. Ferguson, chairman of the executive committee, and chief of staff : "There is an Eastern adage that when the house is finished the master dies. Nothing is to be so dreaded, said the early Egyptians, as a hope realized. The dreamy but searching fancy of the ancient East finds color of fact in the death of him whose loss we deplore today. He was in truth the master builder of this institution. And we recall that only a few short weeks since he told us that his builder's task was practically completed; that the house was finished—the foundations deep and lasting, the superstructure solid and stable, the idea behind the fact lofty and imperishable. The work of course was to go on; there must be further growth, enlargement, branching out. But these were in the nature of additions—the main fact and its assured perpetuation were accomplished. And as it had all been the joy of his strength, the pride of his life, the glory of his advancing years, the "baby of his old age," as he himself whimsically declared—surely in this his hope had been realized; and then, as the adage says, came death. Was it untimely? Who shall dare to aver it? It came to him, as every one who glories in the splendid struggle of life would have it come—in the saddle, with lance at rest, with visor down, with every pulse and sinew and nerve enlisted in the fray.

'Obeying the voice at eve obeyed at prime.'

"This unflagging zeal, this scorn of rest and of the allurements of pleasure, this passionate energy which pushed him into the fighting line at every point made up the motives of his life. 'A few more years—five or six perhaps,' he had said of late, and he would step aside—the calm of retrospect to displace the tireless labor which had spanned his life. We may well believe now that knowingly and purposely he named a period well in advance of the expected hour when

'The God of bounds
Who sets to seas a shore,
Should come to him in his fatal rounds
And say "No more."

"Death never becomes a commonplace. The mystery of all time which no genius has ever fathomed, the misty curtain which no human eye has pierced—the righteous man can meet them unmoved for himself, but not for his friends, not for those he loves. The death of those we cherish is one of the few absolutely irreparable losses which humanity is called upon to bear. In the death of this big, brave, strong man, with his instincts for leadership, his splendid intellect, his great professional ability, his rugged character, his undaunted courage, the directors of this institution are conscious of a loss which to many may well appear irreparable. To individuals that will be so indeed. But, as we know, to ideas a broader law applies. As he himself declared, this great work, of which he was the founder, must go on; time and the inevitable law of healing shall work their way; and from the ashes of this man's noble conception there shall develop and become perpetuated a splendid memorial to his life-work, dearer we believe to him than any eulogy.

"No further seek his merits to disclose,
Nor draw his frailties from their dread abode.
There they alike in trembling hope repose,
The bosom of his Father and his God."

*New York Medical Journal, Sept., 22, '06.*

---

CÆSARIAN SECTION IN THE TREATMENT OF PLACENTA PREVIA.

Briggs reaches the following conclusions in the *Journal of the American Medical Association* of May 12, 1906:

Every pregnant woman should be examined during the sixth month to determine the presence or absence, and, if present, the degree, of placenta previa. The examination should be made bimanually both by vagina and by rectum, and stethoscopically by the vagina and by the abdomen.

In case of central previa, elective Cæsarian section of the Sänger type should be done at the moment of greatest viability of fetus compatible with least danger to the mother.

In case the fetus is dead and labor does not set in spontaneously, it should be induced after placental circulation is shut off.

In emergency cases, when the patient is not exsanguinated and a sufficiently experienced operator is at hand, the Sänger-Cæsarian section with presumably clean or superficially infected uterus should be done, and the Porro-Cæsarian operation if the uterus be positively and deeply infected. (*A*) In case of total placenta previa with (1) undilated and undilatable cervix; (2)

cancerous or fibroid cervix, pelvic tumors, pelvic contraction, or other obstacle to the usual obstetric procedure; (3) ruptured sac with escape of amniotic fluid, and presenting but undescended head. (*B*) In cases of lateral placenta previa with living child, uncontrollable bleeding, and either (1) undilatable cervix or other obstacle to the indicated obstetric procedure, or (2) ruptured and emptied sac with presenting but undescended head.

In elective cases complete and thorough preparation should be made, and the operation systematically planned; distinct functions should be assigned to each assistant.

In imperative emergency cases the surgeon must operate with what may be at hand—scissors, hemostats, needles, thread, antiseptics, ether.

Hemorrhage may be prevented by giving a full dose of ergot hypodermically ten minutes before beginning the operation, compressing the abdominal aorta as soon as the child is delivered, grasping the neck of the uterus low down with both hands and firmly compressing the uterine arteries, and by faradic stimulation of the uterine muscle.

Shock may be obviated and relieved by preventing hemorrhage; by rapid operation; by introducing physiologic salt solution into the colon, connective tissue, blood-vessels, and abdomen; by hypodermic or intravenous injection of adrenalin chloride solution; and by compressing the abdominal aorta.

In the after-treatment purgation should be avoided; colon injections of saline solution, from eight to sixteen ounces, may be given at intervals of from three to eight hours; the bowels may be moved by enemata; Epsom salts, two to four ounces; glycerin, three to six ounces; asafetida mixture, fifteen to thirty ounces.

On the first evidence of uterine infection prompt and energetic measures of local disinfection should be instituted by means of antiseptic exosmosis and drainage.

---

THE possibility of gastric cancer must be considered in cases of supposed pernicious anemia.—*American Journal of Surgery.*

